# Phlebotonics for haemorrhoids

**DOI:** 10.1590/1516-3180.20131314T2

**Published:** 2013-08-01

**Authors:** Nirmal Pereira, Danae Liolitsa, Satheesh Iype, Anna Croxford, Muhhamed Yassin, Lang Peter, Obioha Ukaegbu, Christopher van Issum

## Abstract

**BACKGROUND:**

Haemorrhoids are variceal dilatations of the anal and perianal venous plexus and often develop secondary to the persistently elevated venous pressure within the haemorrhoidal plexus. Phlebotonics are a heterogenous class of drugs consisting of plant extracts (i.e. flavonoids) and synthetic compounds (i.e. calcium dobesilate). Although their precise mechanism of action has not been fully established, they are known to improve venous tone, stabilize capillary permeability and increase lymphatic drainage. They have been used to treat a variety of conditions including chronic venous insufficiency, lymphoedema and haemorrhoids.

**OBJECTIVE:**

The aim of this review was to investigate the efficacy of phlebotonics in alleviating the signs, symptoms and severity of haemorrhoidal disease and verify their effect post-haemorrhoidectomy.

**METHODS:**

*Search methods:* We searched the Cochrane Central Register of Controlled Trials (CENTRAL) in the Cochrane Library 2011 issue 9, MEDLINE (1950 to September 2011) and EMBASE (1974 to September 2011).

*Selection criteria:* Only randomized controlled trials evaluating the use of phlebotonics in treating haemorrhoidal disease were used. No cross-over or cluster-randomized trials were included for analysis and any trial which had a quasi-random method of allocation was excluded.

*Data collection and analysis:* Two authors independently extracted the data and analyzed the eligibility of the data for inclusion. Disagreements were resolved by meaningful discussion.

**MAIN RESULTS:**

We considered twenty-four studies for inclusion in the final analysis. Twenty of these studies (enrolling a total of 2344 participants) evaluated the use of phlebotonics versus a control intervention. One of these twenty studies evaluated the use of phlebotonics with a medical intervention and another study with rubber band ligation.

**AUTHORS’ CONCLUSIONS:**

The evidence suggests that there is a potential benefit in using phlebotonics in treating haemorrhoidal disease as well as a benefit in alleviating post-haemorrhoidectomy symptoms. Outcomes such as bleeding and overall symptom improvement show a statistically significant beneficial effect and there were few concerns regarding their overall safety from the evidence presented in the clinical trials.Only randomized controlled trials evaluating the use of phlebotonics in treating haemorrhoidal disease were used. No cross-over or cluster-randomized trials were included for analysis and any trial which had a quasi-random method of allocation was excluded.

**This is the abstract of a Cochrane Review published in the Cochrane Database of Systematic Reviews (CDSR) 2013, issue 5, Art. No. CD004322. DOI:** 10.1002/14651858.CD004322.pub8 (http://onlinelibrary.wiley.com/doi/10.1002/14651858.CD004322.pub3/abstract). For full citation and authors details see reference 1

**The full text is freely available from:** http://cochrane.bvsalud.org/cochrane/main.php?lib=COC&searchExp=Phlebotonics%20and%20for%20and%20haemorrhoids&lang=pt (this link may be temporary)
